# Supplementary material for: Towards High Efficiency and Rapid Production of Room-Temperature Liquid Metal Wires Compatible with Electronic Prototyping Connectors
Source: Micromachines (Basel). 2023 Dec 12;14(12):2227. doi: 10.3390/mi14122227 (PMC10745818; doi:10.3390/mi14122227)
Supplement: Supplementary file 1 [file micromachines-14-02227-s001.zip › Supporting Information.pdf]

Original system for direct co-extrusion of liquid metal core wires is shown in Figure S1. Extrusion is pull under gravity until it is taken up by a winder than can then pull the material at different speeds. Calibration of the flow rate of liquid metal via a custom syringe pump and polymer via the Wellzoom B is completed separately and then different wire parameters can be chosen based on input volumetric rates and pull speeds (up to 300 mm/s).

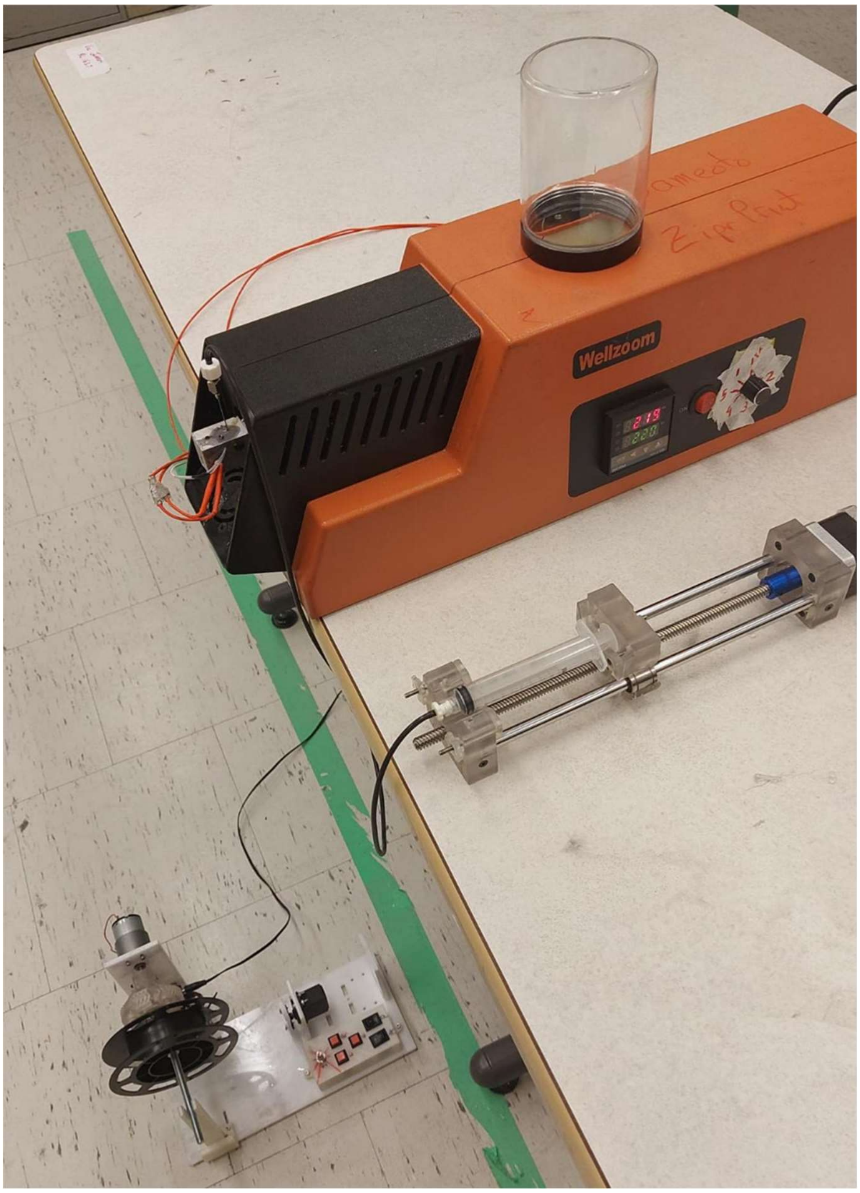

Figure S1 . Photograph of the first-generation co-axial extruder connected to a Wellzoom B and custom syringe pump to control flow of liquid metal.

Figure S2 shows a modified Wellzoom B extruder with an updated co-axial flow system made of brass and capable of more fine positioning and centering of the core. Motor shaft highlighted in red can be directly observed to measure RPM. The system show is set up to have the needle open to atmosphere

for extruding hollow G1645 and G1657 filaments. The needle centering modification is shown in Figure S3.

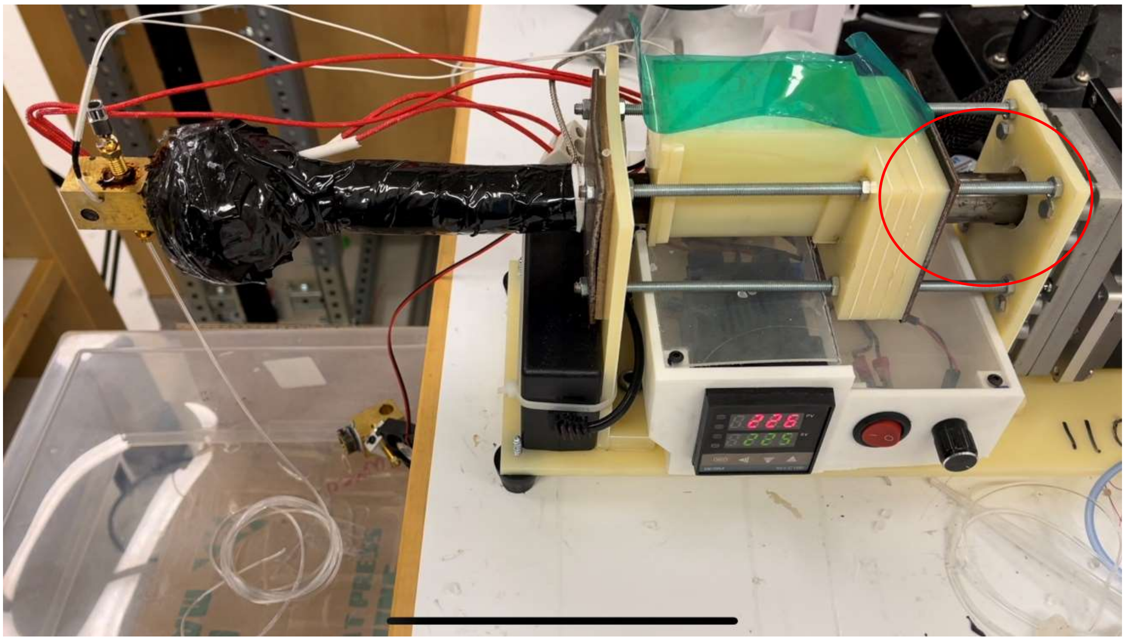

Figure S2 Photography of the modified co-axial extruder used at the University of Alberta. Plastic case is removed to show motor shaft (circled in red) RPM directly and co-axial extruder has a centering feature within the bottom nozzle.

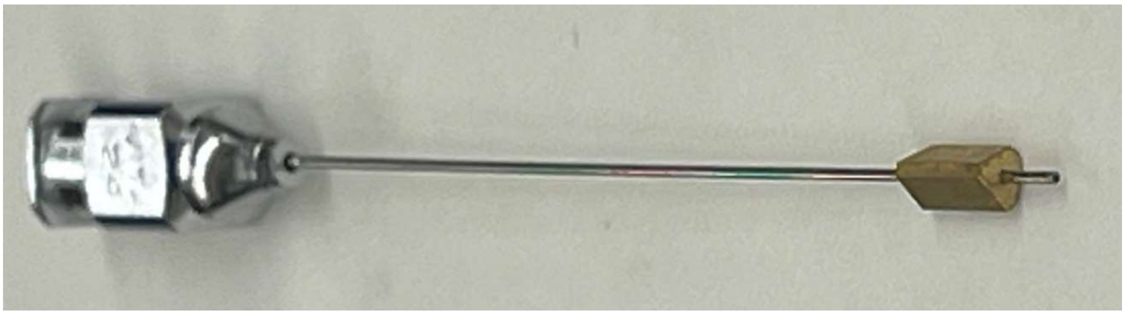

Figure S3 24 Gauge needle with centering element to fit within 3 mm bore nozzle.

When extruding G1657 the overall tube dimensions are smaller due to the lower viscosity for a given temperature of the thermoplastic. Figure S4 shows G1657 and G1645 extruded at the same temperature and drop distance. The smaller core is still possible to have a Dupont connector fit into it but provides a lower ratio of metal to polymer in the tube after filling.

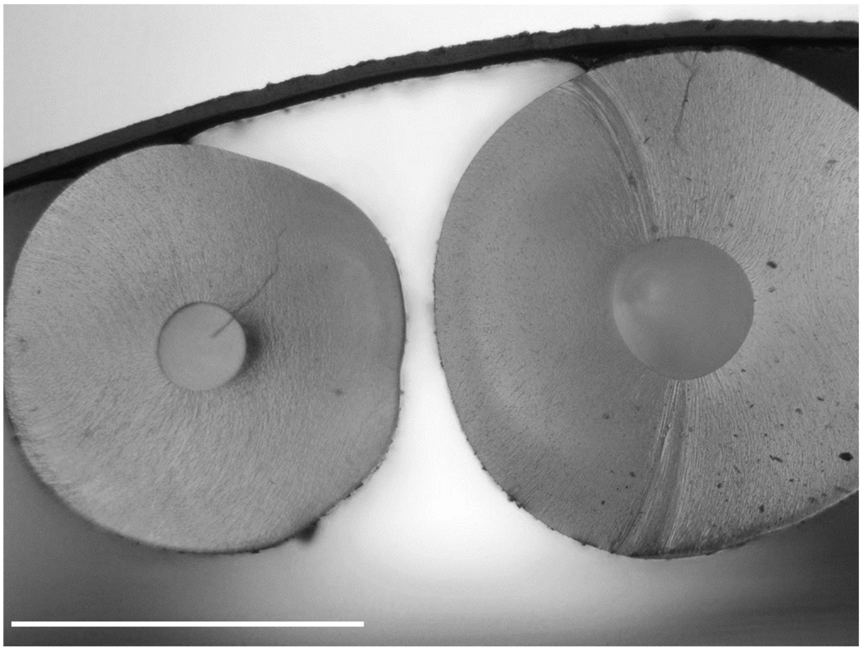

Figure S4 Cross-section view of G1657 (left) and G1645 (right) extruded with the same equipment. The higher viscosity G1645 remains as a larger cross-section with an outer diameter ~1.8 mm and inner diameter ~0.5 mm while the G1657 shows an outer diameter of ~ 1.4 mm and inner diameter ~0.32. The relative internal volume is reduced in this case compared to G1645. White bar represents 1 mm.

Microscope images of alternative sealing/separation techniques of heat sealers or tweezers are shown in table S1. All can be functional but are considerably less reliable than using an ultrasonic blunted knife. Substantial metal oxide residues in the formerly filled tube are seen in the impulse sealer separated channels.

Table S1: Microscope images of alternative EGaIn tube sealing/separation techniques that were attempted.

**Heat and thin tweezers**	**Impulse sealer – pressure then heat**	**Impulse Sealer – heat and pressure**
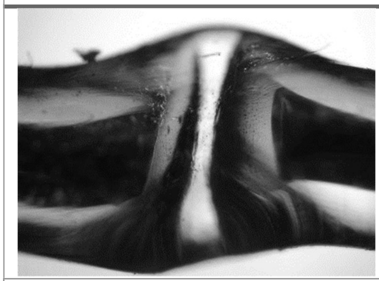	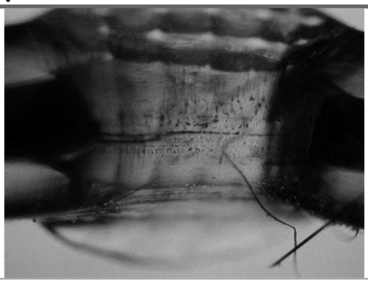	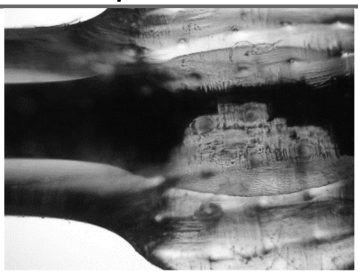
Clean separation, but difficult operation and can	Substantial oxide residue remains	EGaIn not fully disconnected

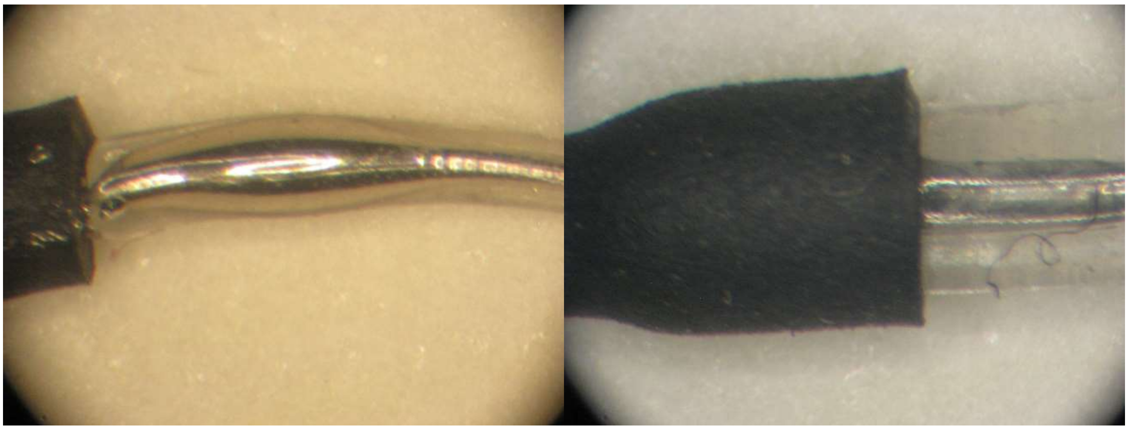

Figure S5: Microscope images of shrink wrap tubes over liquid metal core wires. If the shrink wrap tube is over liquid metal, it will push it out and create distortions in the cross-section or possibly electrically disconnect the system as shown in the left image. If the shrink tube is over the solid metal insert then the same deformations are not seen.

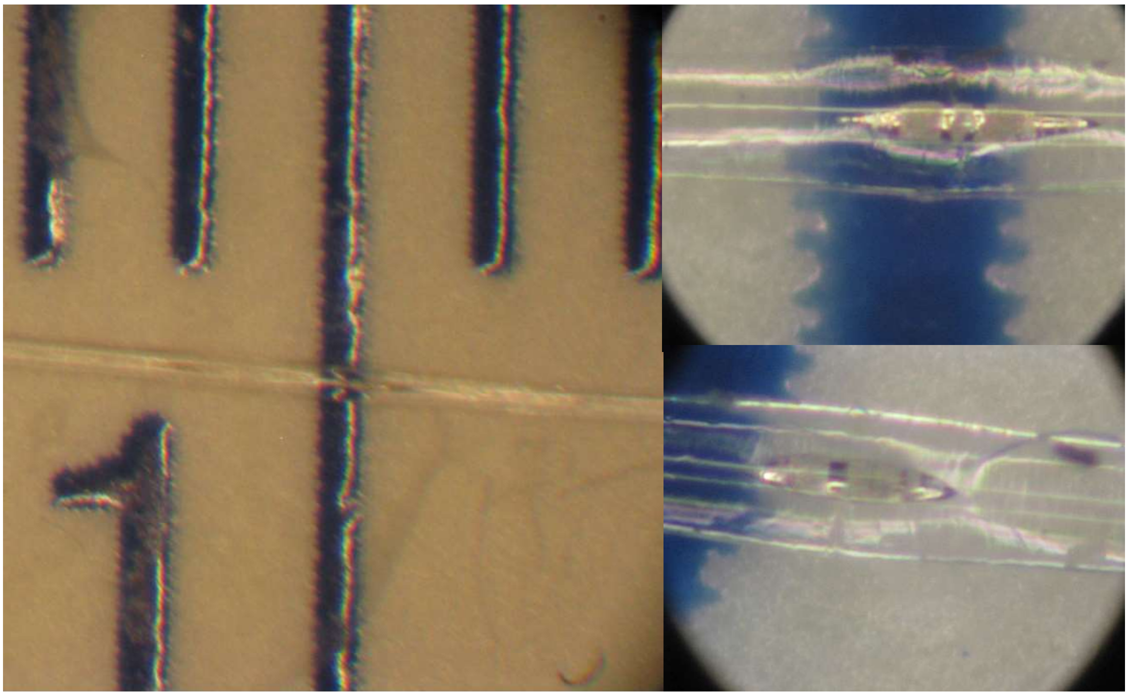

Figure S6: Microscope images pre-filled liquid metal wires drawn out too fast and too hot. Individual droplets of metal rather than a continuous stream are shown in the zoomed in images.

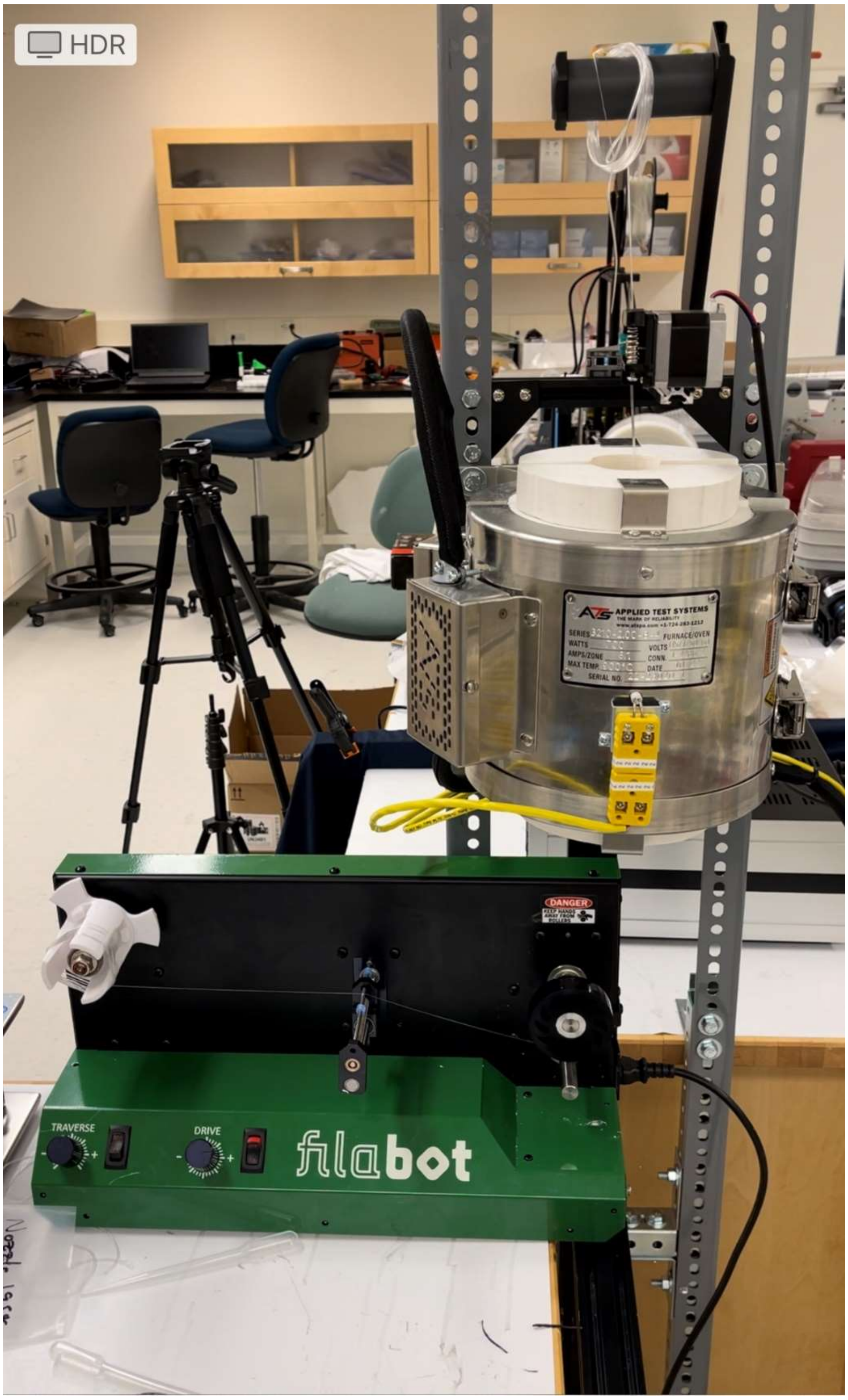

Figure S7: Experimental setup for larger scale production of draw down liquid metal wires. Preformed filaments or direct extrusion into the furnace are options for future developments.

Fabrication and testing of R3VAMP actuators

The base R3VAMP actuators consisted of a tessellated rectangular infill structure inside of an airtight sleeve. When a vacuum pressure was applied, the rectangular infill collapses, retracting the actuator. The infill was printed from white polypropylene filament on an Ender 5 Plus FDM machine. Printing was performed with a nozzle size of 0.4mm and at 220C on a 60C bed of UHMWPE Tape. The total dimensions of the R3VAMP actuator was 10mm x 100mm x 40 mm, and the dimensions of each unit cell was 8mm x 20mm. Polypropylene was selected due to its high compliance and lower stiffness in comparison to PLA, which is more rigid and permanently deforms more easily after repeated cycles. The infill structure was sealed inside of a 2-mil thick polyethylene sleeve using an impulse heat sealer, and an airtight fitting for a silicone tube was installed.

To integrate strain sensing capabilities into the sample actuator, a liquid metal wire was fixed across one face of the actuator such that it would be elastically stretched during actuation, changing its resistance. Various considerations were made to ensure proper functioning of the wire. First, the wire was attached to the actuator while it was in its fully collapsed position. This allowed the wire to be installed in a pre-stretched configuration, allowing it to be stretched taut whether the actuator was in its relaxed or flexed position. To amplify strain, the wire was doubled back so that it spanned the length of the actuator twice. It was also necessary to prevent the wire from making any sharp turns, as this would risk discontinuities. To address this, the wire was first fixed to a set of small bearings, which were then glued to the outer sleeve of the R3VAMP using cyanoacrylate superglue.

To test the R3VAMP, the wire was then connected to a multimeter. A 200g weight was hung from the system, and a vacuum pressure was applied at -10kPa, -20kPa, -30kPa and -40kPa.
